# Comparison of the mitochondrial genomes of three geographical strains of *Apis laboriosa* indicates high genetic diversity in the black giant honeybee (Hymenoptera: Apidae)

**DOI:** 10.1002/ece3.9782

**Published:** 2023-02-01

**Authors:** Xiang‐You Tang, Yu‐Xin Yao, Yao‐Hui Li, Hua‐Li Song, Rui Luo, Peng Shi, Ze‐Yang Zhou, Jin‐Shan Xu

**Affiliations:** ^1^ College of Life Sciences Chongqing Normal University Chongqing China; ^2^ Key Laboratory of Conservation and Utilization of Pollinator Insect of the Upper Reaches of the Yangtze River Ministry of Agriculture and Rural Affairs Chongqing China

**Keywords:** *Apis laboriosa*, honeybee, mitochondrial genome, phylogenetic relationship, subspecies

## Abstract

*Apis laboriosa* is the largest honeybee that lives mainly on cliff faces, with strong migratory ability. In this study, we firstly sequenced and assembled two complete mitochondrial genomes of *A. laboriosa* isolated from two distant locations in China (Chongqing and Shangri‐La regions). Combined with the published mitochondrial genome of *A. laboriosa* from Nepal, comparative genomic analyses were conducted to gain insight into the genetic diversity of giant honeybees from different geographical distributions. The mitochondrial genomes of *A. laboriosa* from Chongqing and Shangri‐La regions were 15,579 and 15,683 bp in length, respectively, both larger than that from Nepal with the length of 15,510 bp. Three mitochondrial genomes all harbor 37 common genes and present the same AT bias and the frequency of codon usage. However, the fragments including *COX*1, SSUrRNA, LSUrRNA, and the AT‐rich region of the mitochondrial genome from Shangri‐La region demonstrate distinctive insertions and deletions compared to those from Chongqing and Nepal regions. Phylogenetic trees of mitochondrial genomes show that *A. laboriosa* from Chongqing is most closely related to that from Nepal, rather than to Shangri‐La. Genetic distance between Shangri‐La and Chongqing or Nepal was even larger than that between the various subspecies of *Apis mellifera*. Overall, these results unmark that *A. laboriosa* in different geographical distributions can exhibit high genetic diversity at the mitochondrial genomic level, and therein, *A. laboriosa* from Shangri‐La may be the subspecies. All these studies will contribute to our understanding of the geographical distribution and genetic differentiation of black giant honeybee in Asian region.

## Graphical abstract

Overall, this study demonstrates that *Apis laboriosa* with different geographical distributions have rich genetic diversity at the mitochondrial level, where *Apis laboriosa* from Shangri‐La even reached the subspecies differentiation level. Meanwhile, our study also found that geographical distance is not the main reason for genetic differentiation, which provides a reference for further expanding research on the geographical distribution of *Apis laboriosa* and analyzing their mechanisms of genetic differentiation.

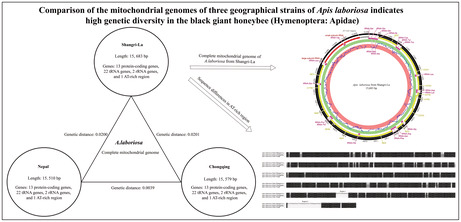

## 1 INTRODUCTION

The mitochondria have been widely applied in studying the organism's evolutionary origin and genetic diversity due to its fast evolutionary rate, simplified structure, and effective genetic information (Sun et al., 2010; Wang et al., 2013; Wolstenholme, 1992). As an important pollinating insect, worker bees in honeybee colony descend from one exclusive queen and matches the mitochondrial matrilineal pattern, which were considered as one of the ideal model organisms for studying genetic diversity based on mitochondrial DNA. Previous researches have found that mitochondrial DNA was differentiated among honeybees of species, subspecies, and geographical groups and can be used to explore the genetic diversity and evolution of honeybee (Cornuet & Garnery, 1991; Franck et al., 2000; Hall & Muralidharan, 1989; Smith et al., 1991; Yu et al., 2019).

*Apis laboriosa* is an important pollinator species usually discovered in the Pan‐Himalaya Mountains in southeastern Asia (Smith, 1871). This insect has a high resistance to adversity and strong collection ability, which are features of great economic and scientific value (Batra, 1996; Woyke et al., 2003). However, due to live on inaccessible cliff faces and strong migratory abilities, it is hard for people to collect samples and implement the scientific research. Up to date, a few studies involved in biology behaviors and morphological comparisons have been conducted on black giant honeybees, which will contribute to the taxonomic classification of *A. laboriosa* comparing with other honeybee species (Cao et al., 2012; Kuang et al., 1983; Lo et al., 2010; Underwood, 1990; Woyke et al., 2008). However, there is no information about morphological comparisons and genetic diversity about intraspecies of this honeybee. Recent study has disclosed the complete mitochondrial genome and whole genome of *A. laboriosa* (Lin et al., 2021; Takahashi et al., 2018), which offers the reference to deeply studying the genetic diversity of *A. laboriosa*.

Previous reports have shown that populations of *A. laboriosa* have declined in some countries and regions due to habitat destruction, human activities, and bee diseases (Allen et al., 1990; Joshi et al., 2004). Therefore, in‐depth research on the genetic diversity of *A. laboriosa* is conducive to the protection and utilization of the species' scarce resources. In this study, we collected *A. laboriosa* samples from Jinyun Mountain in Chongqing and Shangri‐La in Yunnan province to sequence their mitochondrial genome. After the acquisition of the complete mitochondrial genomes of the honeybees from Chongqing and Shangri‐La using Sanger sequencing and assembly. comparative genome analysis was performed to systematically explore the genome variation of *A. laboriosa* from three geographical origins, and then might be helpful to give insight into geographical distribution and genetic differentiation of this extraordinary giant honeybee.

## 2 MATERIALS AND METHODS

### 2.1 Sample collection

Two giant honeybee colonies of *A. laboriosa* was discovered in Jinyun Mountain, Chongqing (29.62°N, 106.28°E), and Shangri‐La, Yunnan (28.56°N, 100.35°E), as shown in Figure 4c, respectively. The 10 adult workers for each colony were collected as samples and then stored in 75% ethanol at 4°C.

### 2.2 Determination of mitogenome sequences

One worker from each colony was randomly selected and total DNA was extracted from the head and chest muscles by CTAB (cetyltrimethylammonium bromide) method (Shi et al., 2020). Six pairs of specific primers covering the entire sequence of the reference mitochondrial genome were designed for synthesis using NCBI Primer‐BLAST tools (https://www.ncbi.nlm.nih.gov/tools/primer‐blast). The details of the primer sequences used in this study were summarized in Table 1. PCR amplification was carried out using a total reaction volume of 20 μL, which consisted of 1 μL (10 μM) forward and reverse primers, 200 ng template DNA, 0.5 μL (1.25 U/μL) Tks Gflex DNA polymerase, 10 μL 2× Gflex PCR Buffer, and 20 μL ddH_2_O. The following PCR procedure was used: a predenaturation step at 94°C for 3 min, followed by 45 cycles of 10 s at 98°C, annealing for 15 s at 58–60°C, then 5 min at 68 °C, and final extension for 10 min at 68°C. After the DNA electrophoresis, the PCR target products were retrieved and sent to Sangon Biotech for DNA sequencing.

**TABLE 1:** Primers used in this study.

Primers	Nucleotide sequence (5′–3′)
AL‐F1	GGACATGTTTTTGATAAACAGGTGAATAATCATTTTGCCG
AL‐R1	GGAAAGATAAATGTAAGTGCCAACAATAGCG
AL‐F2	CAAGTCTCTCTAATTAGATATAAAAAGCCGCC
AL‐R2	GGAATTAATCAATTTCCAAATCCGCC
AL‐F3	GAATCATCAAAGAAAATTCCAAGATTAATTTATTACGTAATTTC
AL‐R3	CAATTTGGGAGATTAAACCAAATCCTGGAAG
AL‐F4	GACCCGGAACAGGATGAACAGTATACCCAC
AL‐R4	CAATATTATTTGCAGGATTAACAGCAAATTTTGAATTAG
AL‐F5	GAATGAACTAAAGAAGATACTGGTGTTGGAGCTATTATTGC
AL‐R5	GTTCTAGAAAGTGTACAAATCGCCCGTCAATTTCATTATTAAG
AL‐F6	CTTGAATTCCATTTATATAATGTATAAATCTCCCAACAC
AL‐R6	GGATAGTGACTATTATTTTAAATCGAGTTGGAGATTC

SeqMan was used to assemble the sequencing results and then achieved complete mitochondrial genomes were annotated using MITOS (http://mitos2.bioinf.uni‐leipzig.de/index.py). The calibration of gene annotation was performed using Geneious v4.8.4 [32] (Kearse et al., 2012) with reference to the published mitochondrial genome annotation of *A. laboriosa* (NC_036155). The two mitochondrial genomes obtained in this study were deposited to GenBank database with accession OP764682 and OP764683.

### 2.3 Bioinformatic and phylogenetic analyses

Mummer 4 (Marçais et al., 2018) was used for genomic collinearity analysis and determination of single nucleotide polymorphisms (SNPs) and insertion–deletion (indels) among three mitochondrial genomes of *A. laboriosa* from Chongqing, Shangri‐La, and Nepal. The analysis of codon usage frequency and codon usage bias were performed using DNASP 6 (Rozas et al., 2017). Muscle was used for DNA sequence alignment.

For the construction of phylogenetic trees, the published complete mitochondrial genomes of closely related bee species were involved and retrieved from NCBI GenBank database, including *Apis cerana* (NC_014295), *Apis mellifera* (L06178, KJ601784, KX870183, KM458618, and KP163643), *Apis laboriosa* (NC_036155), *Apis dorsata* (NC_037709), *Apis andreniformis* (NC_039709), *Apis florea* (KC170303), and *Bombus ignitus* (DQ870926). After alignment with the complete mitochondrial genome sequences and 13 concatenated nucleotide sequences of PCGs (protein‐coding genes), phylogenetic trees were constructed using IQTREE, MEGA, and MrBayes 3.2 software (Minh et al., 2020; Ronquist et al., 2012), with the bootstrap value set to 1000 replicates and 1000,000 iterations. Also, the p‐distance model of MEGA was used for genetic distance analysis.

## 3 RESULTS

### 3.1 Analysis of mitochondrial genome assembly and annotation

**FIGURE 1:**
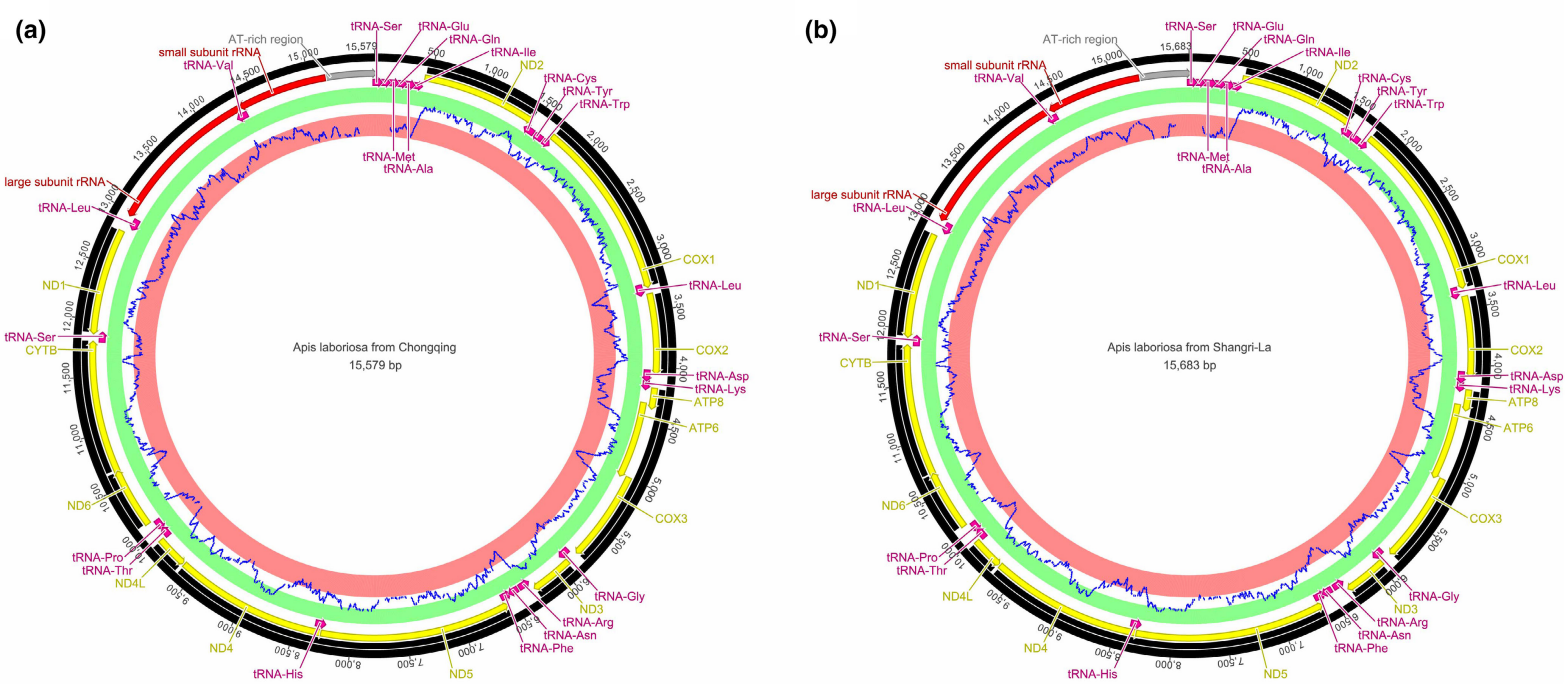
The complete mitochondrial genome of black giant *A. laboriosa* isolated from two regions, including Chongqing (a) and Shangri‐La (b).

As shown in Figure 1, the complete mitochondrial genome lengths of the *A. laboriosa* from Chongqing and Shangri‐La are 15,579 and 15,683 bp, respectively. Both of the genomic lengths were higher than that from Nepal, which was reported as 15,510 bp in length. The A + T contents of all three mitochondrial genomes were 85% (Chongqing), 84.8% (Shangri‐La), and 85% (Nepal), respectively, indicative of obvious AT preference. All three mitochondrial genomes harboring a total of 37 genes, including 13 protein‐coding genes (*COX* 1‐3, *ATP*6, *ATP*8, *ND*1‐6, *ND*4L, and *CYTB*), 22 tRNA genes, 2 rRNA genes, and 1 AT‐rich region (Table 2). However, the mitochondrial genomes differed in the amounts and lengths of intergenic and overlapping regions. The numbers of intergenic region from Chongqing, Shangri‐La, and Nepal are 30, 29, and 30, and accordingly, the lengths of the intergenic regions ranged from 0 to 73, 0 to 73, and 0 to 60 bp, respectively. The numbers of overlapping regions from Chongqing, Shangri‐La, and Nepal are 7, 6, and 7, respectively.

**TABLE 2:** The annotation information of three complete mitochondrial genomes from three different regions (Chongqing/Shangri‐La/Nepal).

Name of gene	Start position	End position	Length/bp	Intergenic nucleotides/bp	Direction
tRNA‐Ser	1/1/1	56/56/56	56/56/56		J/J/J
tRNA‐Glu	62/62/62	126/126/126	65/65/65	5/5/5	J/J/J
tRNA‐Met	141/141/141	206/225/206	66/85/66	14/14/14	J/J/J
tRNA‐Gln	223/244/223	276/315/276	54/72/54	16/18/16	J/J/J
tRNA‐Ala	285/324/285	353/392/353	69/69/69	8/8/8	J/J/J
tRNA‐Ile	357/396/357	426/465/426	70/70/70	3/3/3	J/J/J
ND2	459/498/459	1457/1496/1457	999/999/999	32/32/32	J/J/J
tRNA‐Cys	1457/1496/1457	1522/1561/1522	66/66/66	−1/−1/−1	N/N/N
tRNA‐Tyr	1552/1591/1552	1619/1658/1619	68/68/68	29/29/29	N/N/N
tRNA‐Trp	1639/1678/1639	1705/1744/1705	67/67/67	19/19/19	J/J/J
COX1	1706/1745/1706	3271/3307/3271	1566/1563/1566	0/0/0	J/J/J
tRNA‐Leu	3267/3311/3267	3337/3381/3337	71/71/71	−5/3/−5	J/J/J
COX2	3360/3404/3360	4043/4087/4043	684/684/684	22/22/22	J/J/J
tRNA‐Asp	4042/4086/4042	4112/4156/4112	71/71/71	−2/−2/−2	J/J/J
tRNA‐Lys	4119/4163/4119	4187/4231/4187	69/69/69	6/6/6	J/J/J
ATP8	4194/4238/4194	4352/4396/4352	159/159/159	6/6/6	J/J/J
ATP6	4334/4378/4334	5011/5055/5011	678/678/678	−19/−19/−19	J/J/J
COX3	5019/5063/5019	5798/5842/5798	780/780/780	7/7/7	J/J/J
tRNA‐Gly	5859/5905/5859	5929/5975/5929	71/71/71	60/62/60	J/J/J
ND3	5930/5976/5930	6283/6329/6283	354/354/354	0/0/0	J/J/J
tRNA‐Arg	6319/6366/6320	6388/6435/6389	70/70/70	35/36/36	N/N/N
tRNA‐Asn	6437/6486/6445	6504/6553/6512	68/68/68	48/50/55	J/J/J
tRNA‐Phe	6513/6562/6521	6579/6628/6587	67/67/67	8/8/8	N/N/N
ND5	6584/6633/6592	8248/8297/8256	1665/1665/1665	4/4/4	N/N/N
tRNA‐His	8249/8298/8257	8316/8365/8324	68/68/68	0/0/0	N/N/N
ND4	8252/8301/8260	9607/9656/9615	1356/1356/1356	−65/−65/−65	N/N/N
ND4L	9650/9696/9658	9910/9956/9918	261/261/261	42/39/42	N/N/N
tRNA‐Thr	9923/9969/9931	9988/10034/9996	66/66/66	12/12/12	J/J/J
tRNA‐Pro	10002/10048/10010	10070/10116/10078	69/69/69	13/13/13	N/N/N
ND6	10115/10163/10121	10627/10675/10633	513/513/513	44/46/42	J/J/J
CYTB	10653/10698/10658	11801/11846/11806	1149/1149/1149	25/22/24	J/J/J
tRNA‐Ser	11821/11868/11827	11888/11935/11894	68/68/68	19/21/20	J/J/J
ND1	11890/11937/11896	12807/12854/12813	918/918/918	1/1/1	N/N/N
tRNA‐Leu	12881/12928/12814	12951/12998/12884	71/71/71	73/73/0	N/N/N
large subunit rRNA	12982/13029/12915	14325/14352/14258	1344/1324/1344	30/30/30	N/N/N
tRNA‐Val	14289/14346/14222	14362/14412/14295	74/67/74	−37/−7/−37	N/N/N
small subunit rRNA	14361/14411/14294	15125/15221/15058	765/811/765	−2/−2/−2	N/N/N
AT‐rich region	15126/15222/15059	15579/15683/15510	454/462/452	0/0/0	J/J/J

### 3.2 Codon bias analysis of coding genes

**FIGURE 2:**
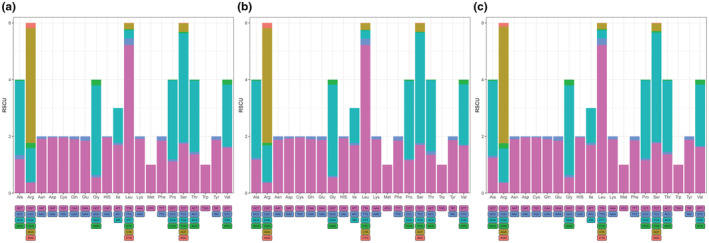
The statistics of codon bias of 13 protein‐coding genes of *A. laboriosa* from Chongqing (a), Shangri‐La (b), and Nepal (c).

We analyzed the frequency and relative synonymous codon usage (RSCU) of 13 protein‐coding genes (PCGs) in the mitochondrial genomes of *A. laboriosa* from Chongqing, Shangri‐La, and Nepal. The RSCU value of the 13 protein‐coding genes are consistent across the three geographical regions and all are >1 (Figure 2), indicating that all codons have biased. NNA‐type codons accounted for 48.23%, 48.41%, and 48.29% of the total number of codons, suggesting that codons with A at the third locus are utilized most frequently.

### 3.3 Analysis of mitochondrial genome structure and sequence variation

**FIGURE 3:**
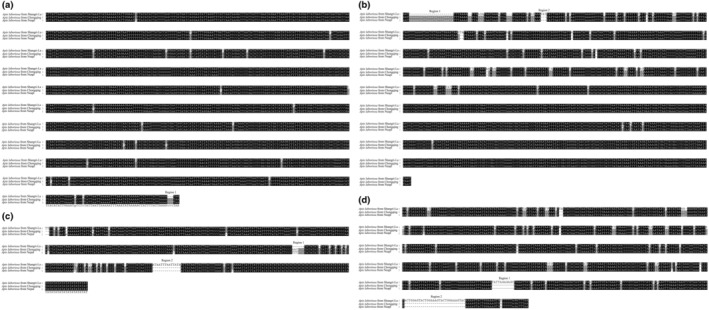
The multiple sequence alignment of *A. laboriosa* genes from three regions. a: COX1, b: Small subunits of rRNA, c: Large subunits of rRNA, d: AT‐rich region.

We conducted a pairwise comparison of SNPs and indel among three genomes to examine the variation of genome structure and sequence. 295 SNPs and 230 indel loci were identified between Shangri‐La and Nepal, 292 SNPs and 161 indel loci were identified between Chongqing and Shangri‐La, nevertheless, only 59 SNPs and 18 indel loci were identified between Chongqing and Nepal. In particular, the genome from Shangri‐La contains more insertions and deletions larger than 2 bp in *COX*1, the small subunits of rRNA (SSUrRNA), large subunits of rRNA (LSUrRNA), and the AT‐rich region based on the alignment of multiple sequences (Figure 3). Previous studies have shown that both AT‐rich region and rRNA are important molecular markers for the taxonomic identification of subspecies (Boore, 1999; Cook et al., 2005; Gao et al., 2011; Li et al., 1995; Rougemont et al., 2004). Therefore, indel fragments of AT‐rich region and rRNA presented in the *A. laboriosa* from Shangri‐La indicate it might be subspecies.

### 3.4 Phylogenetic and genetic distance analysis

**FIGURE 4:**
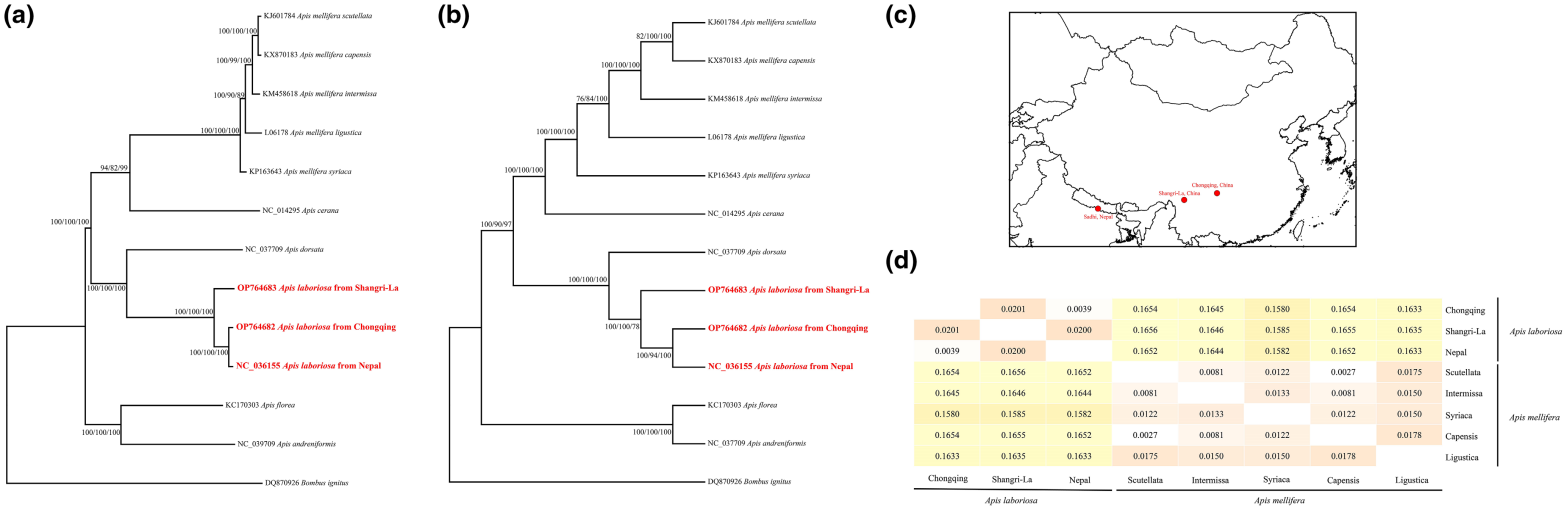
Phylogenetic relationship and geographical location of the black giant honeybees in this study. Bootstrap values present in the above of branch using neighbor‐joining, maximum‐likelihood, and Bayesian‐inference methods. The species *Bombus ignitus* were used as the outgroup. a: full‐length mitochondria, b: 13 PCGs, c: the geographical location of the black giant honeybees, d: pairwise comparison of genetic distance between *Apis laboriosa* and *Apis mellifera*.

Two phylogenetic trees based on the mitochondrial genomes and 13 protein‐coding genes were constructed using three methods including IQTREE, MEGA, and MrBayes (Figure 4). Both trees showed that the *A. laboriosa* from Chongqing, Shangri‐La, and Nepal can be assembled into one group and were sistered to another giant honeybee, *Apis dorsata*. Also, consistent results from two trees showed that Chongqing and Nepal grouped together and form a sister taxon to Shangri‐La, suggesting that the *A. laboriosa* from Chongqing was more closely related to that from Nepal. Pairwise comparison of genetic distance also showed that the genetic distance of Chongqing and Nepal (0.0039) was smaller than Shangri‐La and Chongqing (0.0201), or Shangri‐La and Nepal (0.0200), supporting the same conclusion that the *A. laboriosa* from Chongqing and Nepal have a closer relationship (Figure 4d). Significantly, the genetic distances between Shangri‐La and Chongqing or between Shangri‐La and Nepal, were significantly greater than the genetic distance between various pairwise subspecies of *Apis mellifera* (0.0027–0.0178), as shown in Figure 4d. Based on the results above, we propose that *A. laboriosa* from Shangri‐La may be a geographical subspecies.

## 4 DISCUSSION

Black giant honeybee, *A. laboriosa*, is usually found in the Himalayas and Hengduan Mountains at an altitude of about 1200–3500 m (Underwood, 1990). Due to specific geographical distribution and rocky seclusion (Roubik et al., 1985; Woyke et al., 2012), field observation and sample collection become considerable difficulty compared with other semidomesticated bees within the *Apis* genus. Thus, few studies involved in morphological character, behavior, and genetic difference have been conducted on this unusual honeybee.

In this study, two complete mitochondrial genomes of *A. laboriosa* from Chongqing and Shangri‐La regions were obtained, with the addition of the third published mitochondrial genome of *A. laboriosa* from Nepal (Takahashi et al., 2018), it offers a great opportunity to illustrate the genetic diversity among different regions of *A. laboriosa*. According to our results, the mitochondrial genomes of *A. laboriosa* from Chongqing and Shangri‐La regions were 15,579 and 15,683 bp in length, respectively, both larger than that from Nepal with the length of 15,510 bp. Three mitochondrial genomes all harbor 37 common genes and present the same AT bias and the frequency of codon usage. However, the fragments including *COX*1, SSUrRNA, LSUrRNA, and the AT‐rich region of the mitochondrial genome from Shangri‐La region demonstrate distinctive insertions and deletions compared to those from Chongqing and Nepal regions. Previous studies show that the AT‐rich region is usually responsible for regulating gene duplication and transcription in the mitochondrial genomes of insects, and its sequence length varies considerably among different insect groups and even within genera (Boore, 1999; Cook et al., 2005). Moreover, rRNA is also commonly used for the taxonomic identification of species (Gao et al., 2011; Li et al., 1995; Rougemont et al., 2004). This seems to indicate the special characteristics of the black giant honeybee in Shangri‐La. In addition, both full‐length mitochondria and 13 PCGs trees showed that Chongqing and Nepal grouped together and form a sister taxon to Shangri‐La, and the genetic distances between Shangri‐La and Chongqing or between Shangri‐La and Nepal, were significantly greater than the genetic distance between various pairwise subspecies of *A. mellifera*. Therefore, we speculate that the *A. laboriosa* from Shangri‐La may be a geographical subspecies. Of course, morphological classification will be required so as to provide further evidence for distinguishing subspecies diversification, according to the classic anatomy methods (Mi & Tan, 2007; Ruttner et al., 1978). Due to the limitations in the number of collected bee samples from Shangri‐La and Nepal, morphological analysis cannot be carried forward in this study. Previous study (Cao, 2012) has reported the 38 morphological indices of *A. laboriosa* that isolated from the Baoshan, Yunnan of southwest China, which will offer valuable information for morphological comparison in future.

It is worth noting that the geographical distance between Chongqing and Shangri‐La (588 km) is significantly smaller than the distance between Chongqing and Nepal (2312 km), but the number of SNPs and indel loci between the *A. laboriosa* from Chongqing and Shangri‐La was significantly greater than that between Chongqing and Nepal. It seems that no correlation between genetic diversity and geographical distance in the black giant honeybee base on our results. Previous reports showed that the genetic differences of honeybee species can be influenced by altitudinal factors (Kitnya et al., 2020; Montero‐Mendieta et al., 2019). As we know, *A. laboriosa* are mainly distributed in the Himalayas and Hengduan Mountains at an altitude of above 2000 m (Smith, 1871; Underwood, 1990). However, the location of the black giant honeybee found in Chongqing is only 224 m. Hence, we presumed that the bee colony in Chongqing may be those honeybees that were margined from high altitudinal regions. More samples need to be collected in future to prove our presumptions.

In conclusion, our results unmark that *A. laboriosa* in different geographical distributions exhibits high genetic diversity at the mitochondrial genomic level, and therein *A. laboriosa* from Shangri‐La may approach the subspecies level of differentiation. Although more evidence is still needed to support these inferences.

## AUTHOR CONTRIBUTIONS

**Xiang‐You Tang:** Writing – original draft (equal). **Yu‐Xin Yao:** Formal analysis (equal). **Yao‐Hui Li:** Formal analysis (equal). **Hua‐Li Song:** Methodology (equal). **Rui Luo:** Methodology (equal). **Peng Shi:** Resources (equal). **Ze‐Yang Zhou:** Supervision (equal). **Jin‐Shan Xu:** Project administration (equal).

## CONFLICT OF INTEREST STATEMENT

We certify that there is no conflict of interest with any financial organization regarding the material discussed in the manuscript.

## Data Availability

All the newly sequenced genomic data in this study are deposited in GenBank database with accession OP764682 and OP764683.
